# Are preoperative oral antibiotics effective in reducing the incidence of anastomotic leakage after colorectal cancer surgery? Study protocol for a prospective, multicentre, randomized controlled study

**DOI:** 10.1186/s13063-022-06235-7

**Published:** 2022-05-23

**Authors:** Rui Qi Gao, Wei Dong Wang, Peng Fei Yu, Zhen Chang Mo, Dan Hong Dong, Xi Sheng Yang, Xiao Hua Li, Gang Ji

**Affiliations:** Department of Digestive Surgery, Xi Jing Hospital, Fourth Military Medical University, Xi’an, Shaanxi China

**Keywords:** Antibiotic, Anastomotic leakage, Colorectal surgery, Preoperative preparation, Protocol

## Abstract

**Introduction:**

The optimal preoperative preparation for elective colorectal cancer surgery has been debated in academic circles for decades. Previously, several expert teams have conducted studies on whether preoperative mechanical bowel preparation and oral antibiotics can effectively reduce the incidence of postoperative complications, such as surgical site infections and anastomotic leakage. Most of the results of these studies have suggested that preoperative mechanical bowel preparation for elective colon surgery has no significant effect on the occurrence of surgical site infections and anastomotic leakage.

**Methods/design:**

This study will examine whether oral antibiotic bowel preparation (OABP) influences the incidence of anastomotic leakage after surgery in a prospective, multicentre, randomized controlled trial that will enrol 1500 patients who require colon surgery. The primary endpoint, incidence of anastomotic leakage, is based on 2.3% in the OABP ± mechanical bowel preparation (MBP) group in the study by Morris et al. Patients will be randomized (1:1) into two groups: the test group will be given antibiotics (both neomycin 1 g and metronidazole 0.9 g) the day before surgery, and the control group will not receive any special intestinal preparation before surgery, including oral antibiotics or mechanical intestinal preparation. All study-related clinical data, such as general patient information, past medical history, laboratory examination, imaging results, and surgery details, will be recorded before surgery and during the time of hospitalization. The occurrence of postoperative fistulas, including anastomotic leakage, will be recorded as the main severe postoperative adverse event and will represent the primary endpoint.

**Ethics and dissemination:**

Ethics approval was obtained from the Chinese Ethics Committee of Registering Clinical Trials (ChiECRCT20200173). The results of this study will be disseminated at several research conferences and as published articles in peer-reviewed journals. Protocol was revised on November 22, 2021, version 4.0.

**Trial registration:**

ChiCTR2000035550. Registered on 13 Aug 2020.

**Supplementary Information:**

The online version contains supplementary material available at 10.1186/s13063-022-06235-7.

## Introduction

Preoperative preparation of elective colorectal surgery has been debated in academic circles for decades. In 1973, Nichols et al. conducted a prospective randomized controlled trial of preoperative mechanical bowel preparation (MBP) versus MBP + oral antibiotic bowel preparation (OABP) and published a subsequent retrospective analysis [1, 2]. The results showed that the combined treatment programme can significantly reduce incidence of postoperative complications. Since then, MBP and OABP have become part of preoperative preparation. Over the years, many expert teams have conducted various studies on whether preoperative mechanical bowel preparation and preoperative oral antibiotics can effectively reduce the incidence of postoperative complications, such as surgical site infections and anastomotic leakage. Most of the results of these studies have suggested that preoperative mechanical bowel preparation for elective colon surgery has no significant effect on the occurrence of surgical site infections and anastomotic leakage. On the other hand, preoperative oral antibiotics can effectively reduce the incidence of postoperative complications. Some studies [1, 3, 4] showed no benefit of MBP in open colorectal surgery. Cannon et al. [5] retrospectively studied 9940 patients undergoing colorectal resection from 2005 to 2009 and concluded that preoperative oral antibiotics should be used as part of preoperative preparation for elective colorectal cancer surgery, while the use of preoperative oral antibiotics alone should be validated in randomized trials. A retrospective study conducted in 2017 [6] analysed data from adult patients undergoing elective colorectal surgery between 2012 and 2014 through the NSQIP and concluded that OABP alone could effectively reduce the incidence of postoperative complications. In 2019, B Vadhwana et al. [7] conducted a prospective single-centre randomized controlled study (RCT) to compare the prognostic effects of MBP and MBP + OABP in patients undergoing elective colorectal surgery. They concluded that the preoperative strategy of MBP + OABP could significantly reduce the incidence of postoperative complications and accelerate the recovery of patients. The American Association of Colorectal Surgeons guidelines for bowel preparation in elective bowel surgery published in January 2019 also mentioned that the use of preoperative oral antibiotics alone is a low-grade recommendation based on low-quality evidence that still needs to be validated in randomized trials [8].

Currently, WHO guidelines do not recommend either preoperative oral antibiotics or mechanical bowel preparation alone but require a combination of mechanical bowel preparation and oral antibiotics for elective colon surgery [9]. UK NICE has suggested that preoperative mechanical bowel preparation should not be a part of routine preoperative preparation [10]. In an online survey of surgeons, the European Colorectal Society reported that more than 60% of colorectal surgeries in Europe involve mechanical bowel preparation before surgery, while only 11% of surgeons give patients oral antibiotics before surgery [11]. This situation is quite different from the trends of other domestic and foreign studies mentioned above. At the same time, Chinese research on the correlation between preoperative oral antibiotics and the incidence of postoperative complications is still in its infancy.

To date, there remains a lack of large-sample, multicentre, randomized controlled trial studies on the effect of oral antibiotics before colorectal surgery on the incidence of postoperative anastomotic leakage in the international colorectal treatment field to establish relevant norms for preoperative preparation. Therefore, the aim of this multicentre, pragmatic, adaptive, large-sample RCT is to prove that oral antibiotics alone, compared with no bowel preparation, could effectively reduce postoperative surgical site infection, anastomotic leakage, and hospital readmission in patients undergoing elective colorectal surgery.

## Methods/design

The protocol was structured following the Standard Protocol Items: Recommendations for Interventional Trials (SPIRIT 2013 Checklist) [12] (Appendix S1). The study protocol can be accessed at the chictr.org.cn website (ChiCTR2000035550).

### Study design

**Fig. 1:**
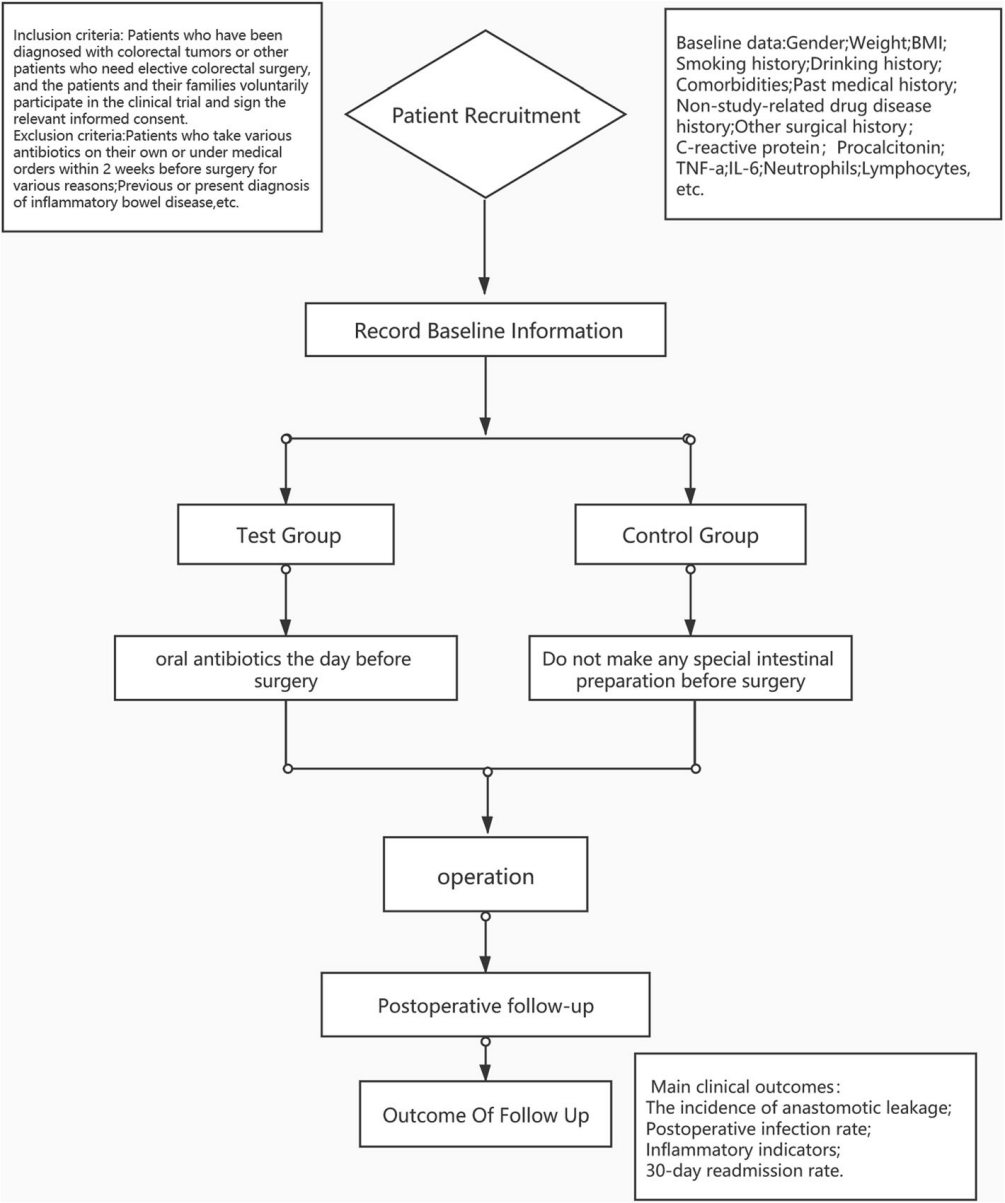
This is the whole flow diagram of the test

This study is a large-sample, multicentre, Superiority RCT in which 1500 patients were randomly assigned to the test group or the control group in a 1:1 allocation ratio. Figure 1 shows the trial flow chart.

### Main objective

By grouping 1500 patients who need colorectal cancer surgery according to the process shown in Fig. 1, we will explore examined whether oral antibiotics alone before surgery could effectively reduce the incidence of anastomotic leakage after surgery.

### Inclusion and exclusion criteria

1. Patients who have been diagnosed with colorectal tumours and are considered for elective surgery (including partial and total resection, with conventional laparotomy and two- or three-hole laparoscopic surgery) and have no obvious contraindications to surgery are considered eligible for this study after the patients and their families agreed to voluntarily participate in the clinical trial and sign the relevant informed consent following a conversation regarding informed consent and the clinical trial.
2. Patients meeting any of the following conditions will be excluded:
  Patients who take various antibiotics on their own or under medical orders for various reasons within the 2 weeks before surgery;
  Patients with previous or present diagnosis of inflammatory bowel disease;
  Patients with a diagnosis of acute intestinal perforation or acute small intestinal diverticulum;
  Patients with ischaemic colitis or infectious colitis included in the diagnosis;
  Patients who require two or more operations at the same time;
  Patients who have been diagnosed with acute or chronic peritonitis or other infectious diseases requiring perioperative anti-infective treatment;
  Patients with any acute physiological disorder indicating that the patient needs emergency, rather than elective surgery [e.g., requires preoperative mechanical ventilation or there is preoperative acute renal failure, preoperative systemic inflammatory release syndrome, sepsis or septic shock, etc.);
  Patients with ASA grade 5;
  Patients with immunodeficiency, immunosuppression or autoimmune diseases (e.g., patients undergoing allogeneic bone marrow transplantation within the last 5 years, taking immunosuppressive drugs, diagnosed with SLE, etc.);
  Patients who refuse to sign informed consent to participate in the trial;
  Patients who are unable to cooperate in a normal fashion with the doctor due to personal reasons or other circumstances in which the investigator considers participation in the experiment unsuitable;
  Patients who require single-port laparoscopic procedures, natural-port procedures, and various new procedures;
3. The terminating study criteria are as follows:
  Patients who violate the principles of treatment after enrolment (e.g., violate the criteria for enrolment and discharge or do not comply with the medication or surgical arrangement for the study duration);
  Patients who were unable to undergo surgery for various reasons after enrolment (reasons will be recorded);
  Patients experiencing nonstudy-related complications such as drug allergies after enrolment;
  Patients who the investigator does not consider appropriate for continuation (reasons for withdrawal need will be documented);
  Patients who develop severe complications or unacceptable adverse reactions; and
  Patients who were unable to undergo elective surgery due to sudden aggravation or other reasons after enrolment.

### Participating entities

This clinical trial is a multicentre study in which the lead unit is the First Affiliated Hospital of Air Force Military Medical University, and participant hospitals include the Second Affiliated Hospital of Air Force Military Medical University, West China Hospital of Sichuan University, the First Affiliated Hospital of Xi’an Jiaotong University, Tumour Hospital of Tianjin Medical University, Zhongshan Hospital Affiliated to Shanghai Fudan University. These six hospitals are the top third-class A hospitals in China. All these hospitals have sufficient experience in the diagnosis and treatment of digestive tract tumours.

### Recruitment

Recruitment will be encouraged by scheduled newsletters to participating centres. Participants will be given free treatment drugs, appointment registration and hospitalization in advance. Appropriate economic compensation, such as transportation subsidies will also be provided.

### Randomization/Assignment of interventions

Randomization was stratified by centre. Once informed consent is obtained, randomization will be performed via a centrally managed database. After receiving the patient grouping information, designated researchers at each centre will prepare trial drugs or placebo for patients according to the assigned group. In this process, only the researchers are informed about the grouping of patients. They will sign a confidentiality agreement and will not participate in any other links of the trial to ensure that they will not have any impact on the study. After collecting all the data, the designated researcher named the two groups A and B. The analyst will analyse the primary and secondary results without knowing the group name. Only after the analysis of the primary and secondary results can complete blindness be eliminated and ineffective blinding events recorded (for example, the study nurse discloses the patient’s specific medication to the doctor).

The nurses provided the patients with oral antibiotics for the experimental group and placebo for the control group in the ward.

### Allocation concealment mechanism

Randomization will be stratified in each centre. The randomization was performed on a centrally managed database once informed consent is obtained.

### Implementation

The sequence will be generated and forwarded by the computer specialist who created the case report form (CRF) online to implement the online database. By doing so, each centre/investigator can randomize their patients and assign them to the respective group of treatments. The date of randomization will be recorded.

### Blinding (masking)

The blind level was set as double-blind. All patients, doctors, data collectors and analysts will be unaware of the grouping of patients.

### Unblinding

If during the trial the subject has not developed a pregnancy or other emergency, Unblinding is performed in accordance with the normal procedure, i.e. after the trial is closed, checking the CRF against the signatures, a level I Unblinding is performed. The grouping of subjects was determined by Unblinding in level I for statistical analysis. Level II Unblinding was performed after statistical closure and statistical judgment had been made. Drug efficacy was evaluated by Unblinding level II to identify the test and control groups. In the event of an SAE, emergency Unblinding is generally not required and can only be performed if the study drug used has an impact on the choice of treatment regimen. Emergency Unblinding required the investigator to notify the study nurse prior to Unblinding, Unblinding based on the information provided by the study nurse about the medications taken by this subject. The investigator must indicate this on the CRF and complete the Unblinding record form.

### Treatment protocols

All eligible patients with colorectal cancer will be randomly assigned to the test group or the control group in a 1:1 allocation ratio. Participants in both groups will undergo similar perioperative procedures with the exception that the test group will receive oral antibiotics before surgery. For the test group, antibiotics will be given (both neomycin 1 g and metronidazole 0.9 g) the day before surgery (at 1 pm, 3 pm and 10 pm). The control group will take the same dose of placebo as the experimental group (Table 1). Intraoperative anal water injection experiments and infiltration by ICG fluorescence were performed to ensure the quality of the anastomosis.

**Table 1:** The medication and usage in this experiment

		Time of drug administration		
Drug name	Dose	13:00	15:00	22:00
Neomycin/placebo	1 g			
Metronidazole/placebo	0.9 g			

### Permitted and prohibited related treatment and care

#### Preoperative management

Inclusion once eligibility has been achieved, surgery should be performed within 2 weeks (inclusive day 14).

After enrollment and until the expected surgical day, if clinical deterioration required the decision to undergo elective surgery as planned based on the judgment of the physician in charge; If emergency surgery was required or if surgery was cancelled, this screening case failed and was not enrolled.

Patients at nutritional risk were allowed preoperative implementation of enteral/parenteral nutrition support.

Perioperative low-molecular-weight heparin prophylaxis, lower extremity antithrombotic pants, aggressive lower extremity massage, and respiratory function training are recommended for high-risk patients who are of advanced age, have smoked, have diabetes mellitus, are obese, have a prior history of chronic cardiocerebral or thromboembolism, and so on. Methods for other potentially high-risk complications were not specified in the study protocol, and decisions were made by the physician in charge at each participating centre based on routine and specific needs of our centre’s clinical practice, but all nonconventional preoperative preparation measures were required to be documented in the CRF.

For patients with a preoperative smoking history, smoking cessation for 4 weeks before surgery is recommended. But this study is not mandated and smoking cessation status and time need to be recorded in the CRF.

For the surgical selection of the procedures performed in this study, the extent of abdominal lymph node dissection, and the method of digestive tract reconstruction were decided by the surgeon in charge of the procedure based on their experience and intraoperative specific circumstances.

Patients' preoperative fasting, water control and other pre-anaesthetic requirements were performed according to the anaesthetic routine protocol of each participating centre, which was not specified in this study.

#### Postoperative management

The observation endpoint was set as 30 days after surgery in this study, and no special requirements were made for postoperative analgesia, fluid replacement, and nutritional support.

### Clinical data

Clinical data from patients will be obtained by medical staff and recorded on an online electronic platform (Http://www.medresman.org.cn) and in the CRF table. The sample will be coded, and the patient’s identity will be known only by the attending physician. The clinical data will include the following: general patient information, past medical history, past surgical history, laboratory examination results, imaging results, surgery details, postoperative infection rate, incidence of postoperative complications, incidence of anastomotic leakage, and 30-day readmission rate after surgery. The timing and processing of the above-recorded contents will all be reflected in the CRF table, and the laboratory examinations will mainly assess preoperative and postoperative routine blood and inflammatory indicators (Table 2).

**Table 2:** The test and data acquisition schedule for this experiment. Intraoperative anal water injection experiments and infiltration by ICG fluorescence were performed to ensure the quality of the anastomosis

Trial flow chart									
Stage		Preoperative		Intraoperative		Postoperative		Unplanned follow-up	
Follow up period	2–3 days	1 day	Intraoperative	1 day	3 days	5 days	14 days	30 days	Unplanned follow-up
Baseline data collected	√	—	—	—	—	—	—	—	—
inclusion and exclusion	√	—	—	—	—	—	—	—	—
Sign informed consent	√	—	—	—	—	—	—	—	—
Group determination	√	—	—	—	—	—	—	—	—
Fill in the basic information	√	—	—	—	—	—	—	—	—
Physical examination	√	—	—	—	—	—	—	—	—
Imaging examination	√	—	—	—	If necessary	If necessary	√	If necessary	If necessary
Blood routine examination	√	—	—	—	√	√	√	If necessary	If necessary
Oral antibiotics	—	√	—	—	—	—	—	—	If necessary
Safety observation	—	—	√	√	√	√	√	√	If necessary
Operational observation	—	—	√	—	—	—	—	—	If necessary
Record adverse events	—	—	√	√	√	√	√	√	If necessary
Other works	√	√	√	√	√	√	√	√	If necessary

A detailed description of the above data is shown in the CRF table.

### Sample size estimate

Due to the lack of international studies on the correlation between the use of oral antibiotics alone and the incidence of anastomotic stoma after surgery, we can only refer to the correlation data between oral antibiotics before surgery and the incidence of total complications in the previous literature to estimate the sample size. Based on the data in the study by Morris et al. [5], the anastomotic leakage rate in this trial was defined as 2.3%, and there is minimal literature available on the comparison between OABP alone versus no preparation. To verify whether OABP is effective in reducing the incidence of anastomotic leakage after colorectal surgery, we designed a superiority test with a superiority margin of 1% (*α*= 0.01, *β*= 0.01, 99% power). With a standard error of 0.01 and a confidence interval of 99%, a sample size of 1232 was needed. To minimize sampling error and account for the rate of loss to follow-up for various reasons, we determined the sample size to be 1500.

### Strategies to improve adherence to protocols

1. Screening: fill in the screening form before enrolment, and attach the gastroscopy, CT, and angiography data.
2. Follow up: telephone/Internet follow-up is allowed. It is recommended that the researcher provide follow-up methods (telephone, micro signal, etc.) to the subjects.
3. Withdrawal/protocol change: it is necessary to collect the reasons for withdrawal/protocol change and the treatment options after withdrawal/protocol change.

### Statistical analysis

Standard descriptive statistics will be used to analyse qualitative and quantitative variables such as relative and absolute frequencies, frequency tables, means, medians, standard deviations, ranges, and quartiles. Association of categorical variables will be performed by two-sample *t* tests or Fisher’s exact test. For continuous variables, Student’s *t* test was used for independent samples, and the Mann–Whitney *U* test was used for normally and nonnormally distributed variables.

A 99% confidence level will be considered appropriate for analysis. The analysis for the primary end-point variable (SSI) will be performed with an intention-to-treat approach. The absolute difference in terms of incidence will be computed, along with 99% confidence intervals, and will be expressed as relative risk or odds ratio, as appropriate for each outcome. Descriptive statistics will also be used to describe the most relevant clinical parameter measurements.

For the secondary variables, a similar approach will be used, using the effect estimate with a 99% confidence interval and relative risk and odds ratio for categorical variables.

Any potential effects related to centres or surgeons will be assessed by means of mixed models. For the primary endpoint variable, a bivariant and multivariant analysis will be performed to identify the potential effects of other variables.

The causes of anastomotic leakage after surgery are very complex, and infection is only one potential cause. If there was no significant difference in the infection rate between the two groups in this study and there was a significant difference in the incidence of anastomotic leakage after the operation, then the result was negative; that is, the difference was not related to whether antibiotics were used before the operation; otherwise, there was a relationship.

### Efficacy assessment indicators

The main efficacy indicator will be the percentage incidence of anastomotic leakage after surgery. Anastomotic leakage will be defined as the breakdown of the connection and subsequent leakage of digestive system fluid from a surgical anastomosis of digestive system structures. If postoperative anastomotic leakage is clinically suspected, digestive tract radiography will be performed to diagnose the leak. Usually, sufficient abdominal drainage is the most effective treatment.

The secondary efficacy indicators are postoperative recovery, as follows: (1) postoperative complications (*n*) at 30 days according to the Clavien–Dindo classification, which includes incisional infection, abdominal abscess, intraperitoneal haemorrhage, anastomotic bleeding, postoperative intestinal obstruction, pancreatitis, pulmonary complications, and other organ complications; (2) inflammation index at 1, 7, and 14 days after the operation; (3) rehospitalization rate within 30 days after operation (days); and (4) incision healing.

### Harms

Adverse events refer to adverse medical events that occur in patients of this clinical trial after receiving the medications. In this study, an adverse event will be considered to the therapy from the time when patients sign the informed consent form to 1 month after the end of treatment. Assessing the nature and determining the severity of adverse events will be conducted in accordance with “expert consensus on diagnostic criteria for postoperative complications of gastrointestinal cancer in China”. To assess adverse events and their causal relationship to therapy, the investigator will evaluate the possible associations between adverse events and trial medications. The following five criteria will be used to determine the results: the time of occurrence of adverse events coincides with the time of administration, adverse events are related to known adverse reactions of the medication, adverse events cannot be explained by other reasons, adverse events disappear after discontinuing therapy and adverse events are reproduced after medication re-administration. The results documented as positive, relevant and possibly related will be deemed adverse reactions. The incidence of adverse reactions will be calculated accordingly. To record, process and report adverse events, the investigator will document any adverse events. Records of adverse events will include a description of adverse events and all related symptoms, time of occurrence, severity, duration, measures taken, the results and final outcomes. The reporting methods and treatment measures for severe adverse events will be classified as severe adverse events if they meet one or more of the following criteria: death, life-threatening (e.g., immediate risk of death), prolonged hospitalization or hospitalization, permanent or severe disability, congenital malformations or defects, some events that have not yet caused death, danger to life or hospitalization; we will also consider severe adverse events perpetrated by a physician if they cause harm to the patient or require medication or surgical treatment to avoid the above situation. For any severe adverse events during the clinical trial, the investigator will file a report of severe adverse events within 24 h and report in writing to the Ethics Committee, the superior authorities. The written report will include the time, severity, duration, measures taken and outcomes of serious adverse events.

### Data collection and observation indicators

Baseline data (collected medical history and demographics, including patient sex, age and contact number; detailed medical history, treatment history, body mass index (BMI); routine blood tests; inflammation index; coagulation test (prothrombin time (PT), activated partial thromboplastin time (APTT), thrombin time (TT), fasting blood glucose (FBG), D-dimer, international normalized ratio (INR)) will be recorded. A premedication imaging assessment test (CT/MRI) including enhanced CT or MRI of the chest, abdomen and pelvis will be completed within 2 days before medication administration. All suspected lesions will be evaluated by imaging tests.

### Follow-up

The follow-up phase will start from the first day after surgery. Patients who do not recover from adverse reactions will be treated and followed up closely until they return to the first level or complete recovery specified in the "expert consensus on diagnostic criteria for postoperative complications of gastrointestinal cancer in China". Follow-up tests include the following: incision condition, RBC count, WBC count, haemoglobin, lymphocytes, neutrophils, eosinophils, CRP, calcitonin, TNF-α, IL-6, and imaging (enhanced CT or MRI of the chest, abdomen and pelvis). In addition, quality of life will also be assessed.

### Patient protection/written informed consent forms

Both parties ensure the protection of the patient’s personal records. Except for documents required by law, patient names are not included in any form in tabular reports, publications or any type of research publication document. Informed consent will be formulated in strict accordance with Chinese laws and regulations. Written informed consent, including all changes made throughout the study, must be preapproved by the Internal Review Board/Independent Ethics Committee before inclusion in the study. Medical staff at each centre will obtain a signature with written informed consent from each patient (if the patient is unable to make their own decision for various reasons, the immediate family will decide on their behalf) prior to any specific activities related to the study. Researchers at each centre will submit and keep original copies of all written informed consent forms signed by patients and provide additional copies to patients or their immediate family members for their records.

### Monitoring of the study

Before the start of the study, the personnel of the project unit will visit the research centre and discuss with the researcher (and/or other research-related personnel) their responsibility for the research programme and the responsibility of the project undertaking unit or representative.

During the study period, the project undertaker or the supervisor representing the project will regularly contact the research centre for a number of reasons including the following: providing information and technical support; establishing randomized grouping as required; confirming that the investigator complies with the study plan, that data on the CRFs are accurately recorded, and that dosage of drugs being used is checked; and carrying out original data analysis (e.g., the data on CRFs are related to the records of patients in the hospital, and the research will compare these with other records). This requires direct access to the original records of each patient (e.g., clinical charts).

The investigators will not have access to data until the study is completed and data are analysed. A data monitoring committee will be established at Xijing Hospital, who will assess the results and safety of the treatments performed at all centres.

Representatives authorized by project undertakers, regulatory departments, and independent ethics committees may visit the centre for inspections, including verifying the original data every half year. The purpose of the inspections of the site and personnel is to systematically and independently examine all research-related behaviours and documents, to determine that these behaviours have been managed and that the data have been analysed, recorded and accurately reported in accordance with the research programme, GCP, ICH guidelines and other regulatory requirements.

### Interim analysis plan

A planned interim analysis of the primary endpoint will be performed when 750 patients are enrolled in the study and the postoperative 30-day evaluation is completed. Interim analyses were performed by an independent statistical team. The statistical team will report the analytical results to DSMC (data and security monitoring committee). The DSMC will have unmasked access to all data and will discuss the results of the interim analysis with the principal investigator at all participating centres at an investigator joint meeting. The decision to continue the study was made at a joint investigator meeting.

Interim analysis will be conducted when study enrollment is 50% complete, or 12 months after formal initiation. This study should be terminated if one of any of the following occurs: enrollment ≤ 30%.

### Data validation

1. Querying missing data: The data management committee will contact investigators should any gaps exist in the CRF.
2. Accuracy: At the end of the follow-up for the primary aim, an independent validator at each centre will check 10% of patients included at the centre (minimum 20 patients), selected by the data management committee, assessing the accuracy of predetermined variables. This will be performed on a dedicated page in the CRF, which can be accessed by the independent validator only. We aim to achieve at least 85% accuracy in the patients selected for validation.

Potential discrepancies will be resolved by contacting the centres. Once the discrepancies are clarified, accuracy assessment at those centres could be repeated for different patients. Continued poor accuracy could lead to centre exclusion or revision of the entire local population of included patients.

### Patient and public involvement

Patients or the public will not be involved in the design, conduct, or reporting or dissemination of our research.

## Supplementary Information

**Additional file 1.** SPIRIT 2013 Checklist.

**Additional file 2.**

**Additional file 3.**

## Data Availability

Datasets used and/or analysed in this study are available from the corresponding authors on reasonable request.

## Abbreviations

OABP: Oral antibiotic bowel preparation
MBP: Mechanical bowel preparation
RCT: Randomized controlled study
NSQIP: National Surgical Quality Improvement Program
WHO: World Health OrganizatioSn
UK NICE: National Institute for Health and Clinical Excellence
ASA: American Society of Anaesthesiologists
CRF: Case Report Form
ERAS: Enhanced recovery after surgery
