# Supplementary material for: Are preoperative oral antibiotics effective in reducing the incidence of anastomotic leakage after colorectal cancer surgery? Study protocol for a prospective, multicentre, randomized controlled study
Source: Trials. 2022 May 23;23:436. doi: 10.1186/s13063-022-06235-7 (PMC9128219; doi:10.1186/s13063-022-06235-7)
Supplement: Supplementary file 2 — Additional file 2. [file 13063_2022_6235_MOESM2_ESM.pdf]

Item No：ChiCTR2000035550

Clinical study on the correlation between preoperative oral antibiotics and postoperative anastomotic leakage

Case Report Form

Completed study: ☐Yes ☐No

Medical institutions undertaking clinical trials: ____________________________

Investigator name: ________________

Recorder name: ________________

Study Initiation Date: □□/□□/**20**□□(D/M/Y)

Study end date: □□/□□/**20**□□

Filling instructions

1. Those who pass the screening shall fill in the formal case report form.

2. The case filling must be accurate and clear. The medical record and this form are the original records and shall not be altered at will. When correcting the errors, draw a horizontal line in the middle of the errors, and the original contents can still be seen. Write the corrected records next to them, and write the name of the researcher and the date of change.

eg: the original record is 8.5, the modified style is 8.5, HHY 2019.7 twenty-nine

3. Fill in this form with black neutral pen.

4. The patient's name Pinyin abbreviation shall be filled in four spaces, the first two letters of the two character Pinyin shall be filled in for the two character name, the first letter of the three character name and the second letter of the third character shall be filled in for the three character name, and the first letter of each word shall be filled in for the four character name.

Examples: Zhang Yue, ZHYU, Li Xinhua, LXHU

5. Internal use of all selected items" ×" tagging.

6. During the test, the adverse event record form shall be filled in truthfully. Record the occurrence time, severity, duration, measures taken and outcome of adverse events. In case of serious adverse events, please report to the clinical trial director, ethics committee, implementer and Drug Administration of the center in time.

Team leader:<br>The First Affiliated Hospital of Air Force Military Medical University	Telephone:<br>Fax:
PI:<br>1. The Second Affiliated Hospital of Air Force Military Medical University<br><br>2. West China Hospital of Sichuan University<br><br>3. The First Affiliated Hospital of Xi'an Jiaotong University<br><br>4. Tumor Hospital of Tianjin Medical University<br><br>5.Zhongshan Hospital Affiliated to Shanghai Fudan University	Telephone:<br>Fax:<br><br>Telephone:<br>Fax:<br><br>Telephone:<br>Fax:<br><br>Telephone:<br>Fax:<br><br>Telephone:<br>Fax:

Safety Supervision Department of the State Drug Administration

(010)68313344-1031

Trial Flow Chart

stage		preoperative		Intraoperation		Postoperative		unplanned follow-up	
Follow up period	2-3 days	1 day		1 day	3 days	5 days	14 days	30 days	
Baseline data collected	√	—	—	—	—	—	—	—	—
inclusion and exclusion	√	—	—	—	—	—	—	—	—
Sign informed consent	√	—	—	—	—	—	—	—	—
Group determination	√	—	—	—	—	—	—	—	—
Fill in the basic information	√	—	—	—	—	—	—	—	—
Physical examination	√	—	—	—	—	—	—	—	—
Imaging examination	√	—	—	—	If necessary	if necessary	√	if necessary	if necessary
Blood routine examination	√	—	—	—	√	√	√	if necessary	if necessary
Oral antibiotics	—	√	—	—	—	—	—	—	if necessary
Safety observation	—	—	√	√	√	√	√	√	if necessary
Operational observation	—	—	√	—	—	—	—	—	if necessary
Record adverse events	—	—	√	√	√	√	√	√	if necessary
Other works	√	√	√	√	√	√	√	√	if necessary

Intraoperative anal water injection experiments and infiltration by ICG fluorescence were performed to ensure the quality of the anastomosis.

General information of patients:

Inspection date: □□/□□/20□□

<table>
<tr><td>Date of birth: □□/□□/□□□□
Age： □□
BMI index: □□.□□
Smoking index: □□□
NRS2002 score: □□□</td><td>blood pressure: □□□/□□□mmHg
Pulse: □□□/min
Heart rate: □□□/min
Breathing: □□□/min
Body temperature: □□□□℃
Charlson Comorbidity Index(CCI):____________</td></tr>
</table>

Patient examination / diagnosis information:

Patient chief complaint:____

Examination / diagnostic results:__________________________________

If it is diagnosed as a tumor, please specify the location, size and number of tumors.

Patient's past medical history

Previous medical history: □No □Yes (please fill in the form below)

<table>
<tr><td>Diabetes:</td><td>|___|Yes |___|No</td><td>COPD:</td><td>|___|Yes |___|No</td></tr>
<tr><td>History of hormone use:</td><td>|___|Yes |___|No</td><td>History of heart failure:</td><td>|___|Yes |___|No</td></tr>
<tr><td>Hypertension:</td><td>|___|Yes |___|No</td><td>If yes, please indicate whether you are taking antihypertensive drugs and the name of the drug:</td><td></td></tr>
<tr><td>Renal failure:</td><td>|___|Yes |___|No</td><td>If yes, do you have a history of dialysis :</td><td>|___|Yes |___|No</td></tr>
<tr><td>History of blood products:</td><td>|___|Yes |___|No</td><td>Neoadjuvant chemotherapy and/or radiotherapy</td><td>|___|Yes |___|No</td></tr>
<tr><td>Oral live anticoagulants</td><td>|___|Yes |___|No</td><td>Whether the tumor has spread and metastasis:</td><td>|___|Yes |___|No</td></tr>
<tr><td>Liver disease</td><td>|___|Yes |___|No</td><td>If there are other combined diseases, please fill in</td><td></td></tr>
</table>

Other surgical history of the patient

Other surgical history: □No □Yes

Please specify: 1.________________________________

2.________________________________

3.________________________________

Investigator signature____________ **Date：** □□/□□/20□□

1.Inclusion criteria

	According to the physical examination and collected medical history, please confirm the following:	Yes	No
1	Patients who have been diagnosed with colorectal tumor or other patients who need colon surgery and have no obvious surgical contraindications	☐	☐
2	Patients and their families voluntarily participated in the clinical trial and signed relevant informed consent	☐	☐
3	The patient can communicate well with the investigator and comply with the requirements of the whole trial	☐	☐
If any of the above answers "no", the subject cannot enter the study.			

2.Exclusion criteria

	According to the physical examination and collected medical history, please confirm the following:	Yes	No
1	Patients who took various antibiotics for various reasons or under the doctor's advice within 2 weeks before operation		
2	Previous or current diagnosis of inflammatory bowel disease		
3	Diagnosis of acute intestinal perforation or acute small intestinal diverticulum		
4	The diagnosis includes ischemic colitis or infectious colitis		
5	Patients requiring two or more operations at the same time		
6	Patients who have been diagnosed with acute and chronic peritonitis or other infectious diseases need to undergo anti infective treatment during perioperative period		
7	Any acute physiological disorder indicates that the patient needs emergency surgery and cannot perform elective surgery [such as preoperative mechanical ventilation, preoperative acute renal failure, preoperative systemic inflammatory release syndrome, sepsis or septic shock, etc.]		
8	Patients with immune deficiency, immunosuppression or autoimmune diseases (such as patients with allogeneic bone marrow transplantation in recent five years, taking immunosuppressive drugs, SLE, etc.)		
9	All single hole laparoscopic surgery, natural channel surgery and various new surgical methods are required		
10	Preoperative mechanical bowel preparation is required by the patient or deemed necessary by the doctor		
11	ASA grade 5		
12	Patients refused to sign informed consent to participate in the trial		
13	Other circumstances in which the patient is unable to cooperate with the doctor normally due to personal reasons or the researcher believes that he is not suitable to participate in the experiment		
14	The patient is participating in other clinical trials		
If any of the above answers "no", the subject cannot enter the study.			

Whether it meets the criteria of this study: ☐Yes ☐No

Investigator signature____________ **Date：**□□/□□/20□□

Preoperative laboratory examination

Date：□□/□□/20□□

Subject		Measured value	Unit	Normal or abnormal has no clinical significance	The abnormality has clinical significance
Blood routine	Red blood cell		10^12/L	☐	☐
	Hemoglobin		g/L	☐	☐
	White blood cell		10^9/L	☐	☐
	Lymphocyte		10^9/L	☐	☐
	Neutrophils		10^9/L	☐	☐
	Eosinophils		10^9/L	☐	☐
	Basophil		10^9/L	☐	☐
Inflammatory index	C-reactive protein		mmol/L	☐	☐
	Procalcitonin		mg/L	☐	☐
	TNF-α			☐	☐
	IL-6			☐	☐

Preoperative imaging examination

Inspection items: ☐MRI ☐CT ☐Ultrasonic □Colonoscopy

The imaging report results:

1 .____

2 .____

3 .____

4 .____

Investigator signature____________ **Date：**□□/□□/20□□

Oral antibiotics 1 day before operation

Drug Name	Name of manufacturer	Specifications	Batch number
neomycin/ placebo			
Metronidazole/ placebo			

		Time of Drug Administration		
Drug Name	Bose	13:00	15:00	22:00
neomycin/ placebo	1g			
Metronidazole/ placebo	0.9g			

Table 1 shows the medication and usage in this experiment.

Investigator signature___________ **Date：□□/□□/20□□**

Fill in within 24 hours after operation

Current medical history and operation

1. Diagnosis of current disease: ____________________
2. Operation date: □□/□□/20□□
3. Resection type: ☐Ileocecal ☐Right hemicolectomy ☐Transverse colon resection ☐Left hemicolectomy ☐Sigmoid resection □Anterior rectal resection □Subtotal colectomy
4. Operation method: ☐Conventional laparotomy ☐laparoscopic surgery ☐Laparoscopy converted to open
5. Surgical incision site: ____________________
6. ASA grade：☐1 ☐2 ☐3 ☐4 ☐5
7. Preoperative intravenous antibiotic time (min before incision) : ______________
8. Duration of operation (min) :________________
9. Intraoperative blood loss (mL) :_________________
10. Incision classification: ☐ clean incision ☐ possibly contaminated incision ☐ contaminated incision

Laboratory examination and routine examination (the first day after operation)

Date：□□/□□/20□□

Subject		Measured value	Unit	Normal or abnormal has no clinical significance	The abnormality has clinical significance
Blood routine	Red blood cell		10^12/L	☐	☐
	Hemoglobin		g/L	☐	☐
	White blood cell		10^9/L	☐	☐
	Lymphocyte		10^9/L	☐	☐
	Neutrophils		10^9/L	☐	☐
	Eosinophils		10^9/L	☐	☐
	Basophil		10^9/L	☐	☐
Inflammatory index	C-reactive protein		mmol/L	☐	☐
	Procalcitonin		mg/L	☐	☐
	TNF-α			☐	☐
	IL-6			☐	☐
Routine item	Blood pressure		mmHg	☐	☐
	Pulse		/min	☐	☐
	Heart rate		/min	☐	☐
	Temperature		℃	☐	☐

Investigator signature____________ Date: □□/□□/20□□

Postoperative follow-up records

General information

Subject		D2	D3	D4	D5	D6	D7	D8	D9	D10	D11	D12	D13	D14
Routine item	Blood pressure (mmHg)													
	Pulse (/min)													
	Heart rate (/min)													
	Temperature (℃)													
Incision situation	red and swollen													
	Pain, tenderness													
	Purulent secretion													

Laboratory examination

Subject		D1	D3	D5
Blood routine	Red blood cell(10^12/L)			
	Hemoglobin(g/L)			
	White blood cell(10^9/L)			
	Lymphocyte(10^9/L)			
	Neutrophils(10^9/L)			
	Eosinophils(10^9/L)			
	Basophil(10^9/L)			
Inflammatory index	C-reactive protein(mmol/L)			
	Procalcitonin(mg/L)			
	TNF-α			
	IL-6			

The imaging report results ☐CT ☐Ultrasonic

1.____

2.____

3.____

Investigator signature___________ **Date：** □□/□□/20□□

Adverse Event

Try to use the name of diagnosis rather than the name of symptoms to record the name of adverse events, especially note that different infections should be recorded in detail.

If it is a serious adverse event, please fill in the serious adverse event report form

AE report form adverse event ☐Yes ☐No

adverse event	Date (D/M/Y)	Severity	Action taken (optional)	Related to the present study	result
		1. Mild<br>2. Moderate<br>3 severe	1. Take corresponding treatment measures<br>2. Reoperation<br>3. Withdrawal from the study<br>4. No action taken	1. Related<br>2. Unrelated	1. Recovery<br>2. Remission<br>3. Stabilization<br>4. Aggravation
	Start date: □□/□□/20□□<br>End date: □□/□□/20□□<br>If still present, current date: □□/□□/20□□<br>**Duration**: □□D□□H	□	□、□	□	□
	Start date: □□/□□/20□□<br>End date: □□/□□/20□□<br>If still present, current date: □□/□□/20□□<br>**Duration**: □□D□□H	□	□、□	□	□
	Start date: □□/□□/20□□<br>End date: □□/□□/20□□<br>If still present, current date: □□/□□/20□□<br>**Duration**: □□D□□H	□	□、□	□	□

Mild: adverse effects were mild and did not require medical intervention; Moderate: adverse effects were mild, patient tolerated and required medical intervention; Severe: adverse effects are severe and intolerable by patients, requiring discontinuation and medical intervention.

Investigator signature____________ **Date：** □□/□□/20□□

Serious adverse events

SAE must notify study responsible unit in telephone / network / fax form within 24 hours

SAE report from   Serious adverse events   ☐Yes   ☐No

Types of reports: ☐First ☐Follow up ☐Summary			Date: □□/□□/20□□
Name of medical institution and specialty:			TEL:
Reporting unit:			TEL:
Name of test drug:			Operation name:
Drug type:			Incision classification:
Subject information	Initials:	Gender: ☐Male ☐Female	Birth date: □□/□□/□□□□
	Diagnosis:		Nationality:
SAE condition: ☐Death ☐Rehospitalization or prolonged hospitalization ☐permanent disability / dysfunction ☐Others, please specify____________			
SAE	Start date: □□/□□/20□□	End date: □□/□□/20□□<br>Still have symptoms: ☐Yes ☐No	Duration: □□D□□H
SAE severity: ☐Mild ☐Moderate ☐Severe			
If SAE occurs before operation, please fill in the measures taken for the test medication:<br>☐Continue medication ☐Stop medication			
SAE outcome:<br>☐ Symptoms disappear (Sequelae ☐Yes ☐No)<br>☐ Symptom persistence<br>☐ Death (Time:)			Relationship between SAE and investigational drug use:<br>☐ relevant<br>☐ probably related<br>☐ may be relevant<br>☐ may not be relevant<br>☐ irrelevant
Relationship between SAE and surgery:<br>☐ relevant ☐ probably relevant<br>☐ possibly relevant ☐ possibly irrelevant<br>☐ irrelevant			
SAE domestic reports: ☐Yes ☐No ☐Unknown		SAE foreign reports: ☐Yes ☐No ☐Unknown	
SAE description and treatment details:			

Name of reporting unit: ________________

Title / title of reporter: ________________

Signature of reporter: ________________

Investigator signature______________ Date: □□/□□/20□□

Fill in 24 hours after discharge

Clinical prognostic indicators

1. Whether nosocomial infection has occurred from operation to discharge: □ yes □ no

If yes, please specify the site of infection:___________ Infection time: □□/□□/20□□

2. Surgical incision:

3. Length of stay: □□/□□/20□□

Discharge time: □□/□□/20□□ (please do not fill in if you die in hospital)

4. Whether mechanical ventilation has been conducted in the hospital: □Yes □No

If yes, please record the mechanical ventilation time:

start time: □□/□□/20□□, □□:□□

End time: □□/□□/20□□, □□:□□

5. Have you ever lived in ICU in the hospital: □Yes □No

If yes, please record the time of entering the ICU: □□/□□/20□□, □□:□□

Time out of ICU: □□/□□/20□□, □□:□□ (please do not fill in if you die in ICU)

6. Whether there is excessive organ failure syndrome in the hospital: □ yes □ no

If yes, please record the occurrence time of MODS □□/□□/20□□

Mods score □□

7. Quality of life score: □□□

8. Death in hospital: □ yes □ no

If the patient died, please fill in the date of death: □□/□□/20□□

Whether death is related to disease: □ yes □ no

Whether the death is related to this study: □related □possibly related □possibly unrelated □unrelated

9. Patient's anthropometric index examination date one day before discharge: □□/□□/20□□

Subject	Measured value	Unit
Weight		Kg
Heart rate		/min
Blood pressure		mmHg
Wound healing		

Investigator signature___________ **Date：□□/□□/20□□**

Follow up 30 days after operation

1. Has the patient been rehospitalized within 30 days

If yes, please fill in the rehospitalization time: □□/□□/20□□

Reasons for rehospitalization:________________________________

Treatment:_______________________________

Whether it is related to this study: □ relevant □ possibly relevant □ possibly irrelevant □ irrelevant

2. Quality of life score: □□□

Investigator signature____________ **Date：□□/□□/20□□**

Completion of test

Did the patient complete the clinical trial on schedule?

<table>
<tr><td rowspan="2">☐1.Yes

End date:

□□/□□/20□□</td><td>Test group (optional)</td><td>Control group (optional)</td></tr>
<tr><td>☐1. The patients were followed up for 30 days without abnormality
☐2. The patient completed a 30 day follow-up
☐3. The patient developed anastomotic fistula
☐4. Other complications occurred and were treated</td><td>☐1. The patients were followed up for 30 days without abnormality
☐2. The patient completed a 30 day follow-up
☐3. The patient developed anastomotic fistula
☐4. Other complications occurred and were treated</td></tr>
<tr><td rowspan="2">☐2.No

Study abort date:

□□/□□/20□□</td><td colspan="2">The first requirement to discontinue the test is:
☐1. researcher
☐2. patient
☐3. Sponsor
☐4. Other________________</td></tr>
<tr><td colspan="2">The main reasons for discontinuing the test are:
☐1. adverse event
☐2. Violation of test scheme
☐3. The subject withdrew his decision to participate in the study
☐4. Suspended by the sponsor
☐5. Other________________</td></tr>
</table>

CRF audit statement

As the person in charge of the test center, I hereby declare that all records in this case report form are true, complete and correct.

Center No.: □□

Research Center:_________________________

Signature of center principal:___________________

Date:□□/□□/20□□
