# Supplementary material for: Are preoperative oral antibiotics effective in reducing the incidence of anastomotic leakage after colorectal cancer surgery? Study protocol for a prospective, multicentre, randomized controlled study
Source: Trials. 2022 May 23;23:436. doi: 10.1186/s13063-022-06235-7 (PMC9128219; doi:10.1186/s13063-022-06235-7)
Supplement: Supplementary file 3 — Additional file 3. [file 13063_2022_6235_MOESM3_ESM.docx]

| Supplementary table Operative Characteristics | | |  |
| --- | --- | --- | --- |
|  | Patients, No. (%) | |  |
|  | **Only OABP** | **No bowel preparation** | P Value |
| **Indication for surgery** |  |  |  |
| Colorectal cancer |  |  |  |
| Colorectal adenoma or other benign tumours |  |  |  |
| Diverticulosis |  |  |  |
| Previous volvulus |  |  |  |
| **Resection site** |  |  |  |
| Right side |  |  |  |
| Left side |  |  |  |
| Colectomy |  |  |  |
| **Resection type** |  |  |  |
| Ileocecal |  |  |  |
| Right hemicolectomy |  |  |  |
| Transverse colon resection |  |  |  |
| Left hemicolectomy |  |  |  |
| Sigmoid resection |  |  |  |
| Anterior rectal resection |  |  |  |
| Subtotal colectomy |  |  |  |
| **Surgical approach** |  |  |  |
| Open |  |  |  |
| Laparoscopic |  |  |  |
| Laparoscopy converted to open |  |  |  |
| **Operation details** |  |  |  |
| Preoperative intravenous antibiotic time, min before incision |  |  |  |
| Duration of operation, min |  |  |  |
| Intraoperative blood loss, mL |  |  |  |

Abbreviations: OABP, oral antibiotic bowel preparation.
